# The 2014 Varsity Medical Ethics Debate: should we allow genetic information to be patented?

**DOI:** 10.1186/s13010-015-0028-7

**Published:** 2015-05-20

**Authors:** Kiloran H.M. Metcalfe, Calum A. Worsley, Casey B. Swerner, Devan Sinha, Ravi Solanki, Krithi Ravi, Raj S. Dattani

**Affiliations:** St. Hilda’s College, University of Oxford, Cowley Place, Oxford, OX4 1DY UK; Emmanuel College, University of Cambridge, Saint Andrew’s Street, Cambridge, CB2 3AP UK; St. John’s College, University of Cambridge, St. John’s Street, Cambridge, CB21TP UK; Brasenose College, University of Oxford, Radcliffe Square, Oxford, OX14AJ UK; Trinity College, University of Cambridge, Trinity Street, Cambridge, CB21TQ UK; St. Anne’s College, University of Oxford, 56 Woodstock Road, Oxford, OX2 6HS UK; St Peter’s College, University of Oxford, New Inn Hall Street, Oxford, OX1 2DL UK

## Abstract

The 2014 Varsity Medical Ethics debate convened upon the motion: “This house believes that genetic information should not be commoditised”. This annual debate between students from the Universities of Oxford and Cambridge, now in its sixth year, provided the starting point for arguments on the subject. The present article brings together and extends many of the arguments put forward during the debate. We explore the circumstances under which genetic material should be considered patentable, the possible effects of this on the research and development of novel therapeutics, and the need for clear guidelines within this rapidly developing field.

The Varsity Medical Debate was first held in 2008 with the aim of allowing students to engage in discussion about ethics and policy within healthcare. Two Oxford medical students, Mahiben Maruthappu and Sanjay Budheo founded the event. The event is held annually and it is hoped that this will allow future leaders to voice a perspective on the arguments behind topics that will feature heavily in future healthcare and science policy. This year the Oxford University Medical Society at the Oxford Union hosted the debate

## Background

Over the past decade, research into the therapeutic applications of genetics has grown substantially [1]. The cost of developing new therapies and pursuing new lines of research requires that commercial research companies have some way of recovering costs and making profit from such vast research and development (R&D) expenditure. Patents grant the owner rights to prevent others from selling or benefitting from any product or method that they have invented and these are usually valid for 20 years [2]. In the United States, patents are only granted to those inventions that are deemed novel, non-obvious and that have a clear use (the “utility requirement”) [3]. The granting of a patent requires that the inventor divulge information about the creation of that product, such that others are able to see how the invention is made and how it works [4]. This is distinct from trade secrets, where an inventor or a company must keep the details of the invention to themselves — trade secrets do not prevent independent invention by a third party. UK Law does not allow for the patenting of a “discovery” ipso facto or one where commercial exploitation would be contrary to existing law [4].

In the context of genetics, patents have been sought for a wide range of different “inventions”, most of which result from the isolation and cloning of specific sequences of nucleic acid bases [5]. The utility requirement for a patent to be granted necessitates that these sequences have some function, whether in the form of coding for particular genes, or having some correlation with increased risk of some condition: they cannot simply be random sequences of bases that have been isolated from cells. The standard for utility set by the United States of America (USA) Patent and Trademark Office is that “a person of ordinary skill in the art would immediately appreciate why the invention is useful based on the characteristics of the invention… and the utility is specific, substantial and credible” [6]. In this article we will discuss modern applications of patenting to genetics, such as those for diagnostic tests or treatments utilising genetic vectors. We argue that valid objections to the patenting of genetic products stem must therefore be made on these grounds of utility and novelty requirements, and whether the spirit of these requirements are respected.

### Appropriate patenting

Taken in its widest sense a patent for a gene may be granted for any sequence of bases. Synthetic poliovirus genomes, constructed in vitro from scratch, have been inserted into living organisms [7]; any genetic product constructed in this way could, in theory, be patented. Even if such a sequence of bases is specifically designed (i.e not isolated from a living organism) it is not beyond possibility that a random in vivo mutation could create the same sequence. The issue of patenting parts of living organisms must therefore be borne in mind for all cases of genetic patenting. Objections to genetic patenting on these grounds are based on the claim that nucleic acid sequences are common to all living organisms, and, given that such random mutations can occur, any such patent claim cannot be truly novel.

It would be problematic for the enforcement of the patent if people were able to isolate, literally from themselves, a random sequence of nucleic acid bases and then patent these. Yet in both the UK and Europe the patenting of isolated naturally occurring sequences is still largely permitted [8]. But does the simple isolation of a gene or genetic sequence really merit a patent? We argue that it does not; especially given that the sequence of the human genome is already known and location of the majority of genes have already been identified. Isolation only requires use of simple pattern recognition and long established molecular biology techniques – hardly a demonstration of innovation worthy of a patent.

In keeping with the utility requirement for patentability, most patents concerning genetic isolation are granted to the processes that facilitate the quantification or genotyping of a gene for the purposes of investigating a specific disease. The knowledge gained from genotype studies of Apolipoprotein E (ApoE) variants in the population has been patented for commercial exploitation in a wide variety of conditions, ranging from the role of ApoE in determining the susceptibility to early-onset Alzheimer’s [9], the likely effectiveness of cholinomimetic treatment in patients with Alzheimer’s [10], the predisposition to lipoprotein abnormalities and cardiovascular disease and the predisposition to prostate cancer [11].

A further step in the process, once a gene has been isolated and its role in a disease state quantified, is the use of the gene as a genetic test or screening tool: an application which could in theory be patented. The *Myriad* patent of BRCA1 [12] and BRCA2 [13] is an example of a patent granted for an isolated gene sequence. Certain polymorphisms of the BRCA genes are known to increase risk of breast and ovarian cancer and *Myriad* was the first to successfully isolate and identify these genes. The isolated gene sequence has then formed the basis for a genetic test for the cancer risk increasing forms. However, a recent USA court case, *Association for Molecular Pathology v. Myriad Genetics* [14], overturned the *Myriad* patent and produced a precedent for disallowing the patenting of simple isolated sequences. So while an isolated sequence may have a utility as part of a genetic test, there is a strong argument to say that identification of such a sequence is no innovative and thus not worthy of a patent.

There are, however, circumstances were the utilisation of an existing human genetic sequence can, in our view, fulfil the requirements set out above and therefore form the basis of a patentable commodity. In the case of genetic vectors, the same reasoning can be applied, recognising that there is a significant innovative steps present between the isolation of a nucleic acid sequence and the packaging, delivery and efficacy of such sequence as a therapeutic vector. For example creation of process that delivers a gene via a viral vector requires novel work and more time than simply isolating a gene. The difference between gene therapies and simple gene isolation can be considered due to the technical work involved in creating a new therapy and (perhaps more importantly) the utility of said therapy. Unlike an isolated gene, a therapy is truly novel and has a clear and obvious benefit.

### Current practice and implications of genetic patenting

As mentioned previously, current EU patent law does allow for the patenting of isolated nucleic acid sequences [8]. We can therefore ask whether changing to disallow such patents would actually provide any benefit. In the UK non-compliance is very high and there is little evidence that patent holders take action against those in breach of patent [15]. On the other hand, when a patent is upheld and defended, this has the potential to seriously limit research. Unlike a vector based genetic therapy, which is a ‘finished product’ with a clear use and benefit, gene isolation is the beginning of the research process. If a company successfully patents a specific gene, it could, in theory, then limit all further research into that sequence. While this may be appropriate in a sequence where its role and potential therapeutic benefit has been fully characterised, in other sequences this could have serious consequences. For example, if the TP53 gene sequence (which produces the tumour suppressor protein, *p53*) had been patented and the patent upheld, all research into *p53* and its importance in cancer may have been stopped. A patent allows a company to carry out all the research itself, and in a genetic sequence with many functions (like TP53) it seems very unlikely that all possible functions could be characterised by one laboratory or company. However if taken in its current legal sense this is indeed the case: in *Madey vs Duke*, a case concerning a patented laser, the court ruled that any further research that was “not solely for amusement, to satisfy idle curiosity or for strictly philosophical inquiry” was liable to be in breach of patent [16]. It might therefore be suggested that whilst allowing patents on therapeutic vectors encourages research and innovation by providing a commercial incentive, allowing patents on isolated, naturally occurring sequences is more likely to limit research. The fact that it has not done this so far in the UK is because patents have not been upheld, however there is nothing to prevent a change of attitudes in line with the rapidly changing landscape. Why then are current attitudes averse to pursuing infringements of patents legally? Firstly, it is difficult to definitely prove consequential damages arising solely from research on a nucleic acid sequence when there has been no commercialisation of a further product. Secondly, in practice, commercial companies often stand to gain commercially from a symbiotic relationship with academic researchers who investigate their patented sequence: the researchers gain academic credence and output in the form of publications whist the patent holder gains from knowledge produced at no direct cost to themselves. From a public benefit viewpoint, it is vastly preferable to have patents predominate over the alternative: knowledge stored as a trade secret. Patenting requires that the details of the “invention” are made public. This allows for further research to be carried out by a variety of scientists with different viewpoints, potentially increasing the number of uses of any one nucleic acid sequence as its roles are further characterised.

## Conclusion

An environment needs to exist which allows for both intellectual property rights to incentivise commercial endeavour whilst also being open to collaborative research with the ultimate aim of promulgating new technology for therapeutic benefit. We have argued here that the cautious use of patenting can allow for the creation of new genetic therapeutics, in terms of both convincing the pharmaceutical industry of investing in R&D and providing an open platform of research to the wider scientific community. However, we argue that the current stage at which patents are granted (i.e simply for isolating sequences) in genetics is too early: this is evidenced both on the novelty and utility requirements applied to patents in general, as well as the evidence from current practice and the implications for public benefit.

### About the debate

After a passionate display from both sides, the judges awarded victory to the Oxford team this year. The key area of contention throughout the debate was that of creating an environment which fostered the growth of innovative research in this rapidly developing scientific field. Cambridge, in proposition, argued that well regulated patenting would provide economic incentive to promote developments in the field, which would ultimately provide meaningful therapeutic benefit to the population at large. However, it was Oxford’s view that the patenting of a gene would essentially commodify naturally occurring genetic sequences and that this posed a significant risk to society. Oxford’s main arguments focussed on the fact that patenting naturally occurring sequences did not necessarily require any novel innovation. Oxford argued that, in fact, such patenting might act as a limit on gene research by incentivising companies to simply identify genes rather than gene products.
